# Mathematical coherence in science education: exploring curriculum alignment and student achievement in middle school

**DOI:** 10.3389/fpsyg.2025.1709950

**Published:** 2025-12-10

**Authors:** Semahat Incikabi, Oktay Erbay

**Affiliations:** ^1^Department of Mathematics Education, Faculty of Education, Sinop University, Sinop, Türkiye; ^2^Department of Mathematics Education, Faculty of Education, Hatay Mustafa Kemal University, Hatay, Türkiye

**Keywords:** mathematical coherence in science education, curriculum alignment, middle school achievement, interdisciplinary integration, STEM education

## Abstract

**Objective:**

This study examines how the alignment between mathematics and science curricula affects secondary school students’ science achievement, focusing particularly on the dimension of mathematical consistency in textbook-based test items.

**Methods:**

Using a mixed-methods research design, data were collected from 620 fifth and sixth-grade students in 11 public schools in Turkey. Based on their alignment with the grade-level mathematics curriculum, science questions were systematically categorized as mathematically connected (CCQ) or disconnected (CDQ).

**Results:**

Quantitative analyses revealed that students demonstrated significantly lower achievement on CDQs, indicating that mathematical misalignments in science examinations negatively impacts students’ understanding and performance. Complementary clinical interviews indicated that students often possessed the necessary scientific knowledge but struggled with mathematical reasoning or representations that fell outside the scope of instruction.

**Discussion:**

The findings highlight the importance of deliberate and systematic coordination between the two curricula, particularly in contexts where science and mathematics competencies are assessed concurrently. The study reveals that inconsistencies between curricula place students at a disadvantage in the assessment process and lead to conceptual fragmentation. Accordingly, recommendations have been made to curriculum developers, teachers, and policymakers to strengthen interdisciplinary consistency, increase fairness in assessments, and support students’ science achievement through consistent instructional design.

## Introduction

Textbooks, which are fundamental tools of education, shape the content students encounter and how they learn this content (Chiappetta and Fillman, 2007). In Turkey, the middle school science curriculum has undergone several revisions within past 20 years to ensure that teaching materials align with evolving educational standards and pedagogical priorities (Kurt and Kaya, 2025). These efforts have improved the content and structure of textbooks, necessitating closer examination of the impact of textbook design on learning. Well-designed textbooks that are consistent with curriculum goals and step-by-step structure scientific concepts are known to increase student achievement and maximize “learning opportunities” (Schmidt et al., 2002). Another feature of well-designed books is that they reflect knowledge of other concepts, subjects, and scientific fields, such as mathematics, in a manner appropriate to the student’s level and readiness (Pektaş et al., 2022). In contrast, fragmented or mathematically inconsistent content can hinder conceptual development (Kesidou and Roseman, 2002). Although initiatives such as providing free textbooks in Turkey aim to ensure equal access to quality materials, significant differences persist in the meaningful integration of mathematics in science textbooks (Laçin-Şimşek et al., 2022; Pektaş et al., 2022).

Mathematical complexity and curriculum consistency are two critical dimensions that shape science learning outcomes. Many science questions require students to use mathematical skills such as calculation, data interpretation, or algebraic reasoning; however, when these demands exceed classroom-level competencies, they can create cognitive barriers (Ibrahim et al., 2017; Kuo et al., 2013). Science teachers in Turkey acknowledge the importance of integrating mathematics but report difficulties arising from limited in-service training and insufficient inter-subject curriculum alignment (Kurt and Kaya, 2025). Although reforms have encouraged skill-based and interdisciplinary tasks in recent years, these initiatives can be ineffective without appropriate structuring and curriculum alignment (Ibrahim et al., 2017; Kurt and Kaya, 2025; López-Gay et al., 2015). Effective science teaching depends on the adequacy of mathematical content and consistency across curricula, which enable students to make meaningful connections between scientific ideas. This ensures that the mathematical representations and reasoning used in science align with students’ mathematical proficiency levels (National Research Council [NRC], 2012). However, there is evidence that some Turkish science textbooks are overly comprehensive or fragmented, diminishing their educational value (Cardak et al., 2016).

Globally, science and mathematics educators have long emphasized the necessity of interdisciplinary integration. Leading educational organizations such as the AAAS, NCTM, and NSTA have consistently advocated for the coordinated teaching of science and mathematics (American Association for the Advancement of Science, 1993; National Council of Teachers of Mathematics, 2000; National Research Council, 1996). Numerous studies have shown that mathematical knowledge deficits significantly reduce science achievement, particularly in mathematics-intensive areas such as physics (Basson, 2002; Frykholm and Meyer, 2002; Meltzer, 2002). Large-scale international assessments such as TIMSS also reveal that students tend to perform poorly on science questions that require mathematical reasoning (Wang, 2005). While science education is often based on mathematics, the relationship between the two disciplines is asymmetrical: mathematics can be taught without reference to science, but science, especially the physical sciences, often requires mathematical concepts (Frykholm and Meyer, 2002; National Research Council, 1996). Despite this interdependence, recent research from Turkey points to a curriculum mismatch, where students are expected to solve mathematically complex science problems without prior exposure to the necessary mathematical concepts (Laçin-Şimşek et al., 2022; Pektaş et al., 2022). This lack of coordination between curricula can hinder student learning and place the burden of integration directly on students (Treiner, 2018).

In this context, the present study examines how the alignment or misalignment of mathematical content in science textbooks affects secondary school students’ science achievement. More specifically, it investigates how differences between mathematics and science curricula shape student achievement, as reflected in test items. Accordingly, the study’s fundamental research question is: How does the alignment or misalignment of mathematics and science curricula in test items affect secondary school students’ science achievement?

Although this study was conducted within the centralized educational system of Turkey, its implications extend far beyond the national context. The challenge of ensuring coherence between mathematics and science curricula is not unique to Turkey; it is a recurring issue in many countries where disciplinary boundaries and assessment frameworks remain fragmented. International comparative studies, including TIMSS and PISA, have consistently highlighted similar patterns of misalignment that hinder students’ ability to transfer mathematical reasoning into scientific contexts (Schmidt and Houang, 2007; Wang, 2005). Therefore, the methodological framework and findings presented here offer valuable insights for curriculum designers, teacher educators, and policymakers in diverse educational systems seeking to strengthen interdisciplinary integration through STEM-based approaches. By demonstrating how mathematical alignment influences science achievement, this study contributes to the global discourse on curriculum coherence and the preparation of future-ready learners capable of applying knowledge across disciplinary boundaries.

## Conceptual framework

### Mathematical knowledge in science achievement

Due to their shared focus on the physical universe, science and mathematics are inherently closely linked fields of study. While mathematics offers abstract representations, science presents concrete applications (Basista and Mathews, 2002; McBride and Silverman, 1991). Using scientific content and processes in mathematical problems reinforces the widespread belief that learning becomes more meaningful when mathematical techniques are applied outside of purely mathematical contexts to solve real-world problems (Berlin and White, 2010; Pang and Good, 2000). On the other hand, research shows that using mathematical knowledge and skills in science lessons significantly increases students’ understanding (Bassok and Holyoak, 1989; Kıray and İlik, 2011; Kiray and Kaptan, 2012). Furthermore, many researchers argue that explaining scientific concepts through quantitative mathematical reasoning leads to students developing a deeper understanding (Basista and Mathews, 2002; Park, 2006; Venville et al., 2002).

The moderate correlations between mathematics and science achievement also indicate that strategic mathematical knowledge is necessary for success in science learning (Gustin and Corazza, 1994; Wang, 2005). According to advocates of integrating science and mathematics courses, using mathematical terms and tools facilitates students’ understanding of science concepts (Basista and Mathews, 2002). For example, calculating the pressure exerted by an object on a surface requires knowledge of geometric shapes and surface area. In this regard, it is necessary to make the science program more compatible with the mathematics teaching program to enable students to use and understand mathematics more effectively in science learning.

### Integrated science and mathematics teaching programs

A comprehensive analysis of mathematics and science education trends indicates that calls for interdisciplinary integration are increasing, particularly due to their potential to enhance student engagement (Schroeder et al., 2007). This movement has been supported by important organizations such as the Association for Science and Mathematics in Schools, the National Council of Teachers of Mathematics (NCTM), the American Association for the Advancement of Science (AAAS), and the National Research Council (NRC). It has been reflected in national standards documents such as the National Science Education Standards (National Research Council, 1996) and the National Council of Teachers of Mathematics (1995, 2000). In particular, National Council of Teachers of Mathematics (2000) defined ‘Connections’ as one of the process standards and recommended integration between disciplines such as mathematics and science.

Lederman and Niess (1998) noted that ‘recent reforms have increased interest in curriculum integration, particularly between mathematics and science’ (p. 281). Such calls have led to the development of various theoretical frameworks for integration. For example, Huntley (1998) proposed a continuum model of five categories to define integrated mathematics and science education. This model resembles Lonning and DeFranco's (1997) Continuum Model; it represents the separate teaching of mathematics and science at the extremes and the increasing inclusion of one discipline in the other towards the center. One of the best-known ways of achieving interdisciplinary integration is problem-based learning (PBL) (Savery and Duffy, 1995). However, despite the development of such integrative approaches, their implementation in practice has achieved limited success. Research identifies several interconnected barriers that restrict interdisciplinary (including PBL) in schools. Secondary teachers often perceive themselves as experts in their own disciplines but feel less confident teaching content that crosses subject boundaries (Pang and Good, 2000; Venville et al., 2002). Preparing interdisciplinary lessons also requires significant time and collaboration, which can be difficult to sustain within existing workloads (Clark and Ernst, 2006; Panaritis, 1995; Shoff, 2003). In addition, teachers frequently report a lack of access to appropriate curricular resources and institutional support for integrated instruction (Czerniak et al., 1999; Shoff, 2003). Finally, the traditional, discipline-based organization of curricula and timetables limits flexibility for designing and delivering cross-disciplinary learning experiences (Jacobs, 1989; Morrison and McDuffie, 2009).

Lederman and Niess (1998) observed that “the current reforms have resulted in renewed interest in curriculum integration, especially between mathematics and science” (p. 281). These calls for science and math integration also supported development of numerous frameworks for the integration. Huntley (1998) proposed a continuum framework with five categories for Defining Integrated Mathematics and Science Education. The Mathematics/Science Continuum is similar to the Continuum Model of Integration presented by Lonning and DeFranco (1997), with the ends of both continua representing separate approaches to teaching and learning mathematics and science, and movement towards the middle of both continua representing increased infusion of another discipline (mathematics or science) into the teaching and learning of the other discipline (science or mathematics). Moreover, a large body of literature exists on one potential form of integration, problem-based learning (Savery and Duffy, 1995). Even with specific approaches for interdisciplinary teaching, such as PBL, efforts to encourage integrated instruction have been met with limited success. Four primary factors have been seen to contribute to this. First, secondary teachers see themselves as experts in their own content, but often believe they lack expertise in other disciplines (Pang and Good, 2000; Venville et al., 2002). Second, the time involved in the preparation of interdisciplinary lessons can be daunting (Clark and Ernst, 2006; Panaritis, 1995; Shoff, 2003; Tokmak et al., 2013). Third, teachers feel they do not have access to appropriate curricular resources (Czerniak et al., 1999; Shoff, 2003). The fourth factor that contributes to the limited success of interdisciplinary efforts is the traditional discipline-specific organization of curricula and scheduling (Jacobs, 1989; Morrison and McDuffie, 2009). These difficulties can burden students, particularly when mathematical content is unsuitably integrated into the science curriculum.

Various models have been proposed for curriculum integration. Jacobs (1989) advocated the parallel design of disciplines; in this approach, teachers organize overlapping topics simultaneously without changing the essence of the content. Similarly, Fogarty (1991) presented approaches such as the sequential model, the linked model, and the shared model; if there are common points between disciplines, the content is coordinated. Vars (1991) argued that these methods represent the most fundamental types of integration. However, in countries with centralized curricula, such as Turkey, teachers have limited authority: they can change the order of learning outcomes within a unit. However, they cannot intervene in the order of units. As seen in the study by Pektaş et al. (2022), this situation leads to some science topics requiring mathematical knowledge that has not yet been taught. Therefore, curriculum developers are required to consider knowledge gained from other disciplines when organizing content. While integrative approaches enhance the applicability of mathematics to real-world contexts and interdisciplinary relationships (Berry et al., 2012), they risk undermining the integrity of mathematics curricula. Schmidt and Houang (2007) emphasize that the integrity of the mathematics curriculum is critical to improving student achievement and that mathematics provides a common language for science, engineering, and technology, forming the foundation for other STEM disciplines.

### Integrating science and mathematics education in the case of Turkey

Key indicators of student achievement at national and international levels demonstrate that improvements are still needed in the Turkish education system, particularly to address shortcomings in mathematics and science. In recent years, increasing importance has been placed on developing students’ ability to transfer their mathematical knowledge and skills to new areas such as science and engineering (Flegg et al., 2012; Gill, 1999; Harris et al., 2015). In line with these findings and reformist approaches worldwide, a STEM Education Action Plan focusing on integrating mathematics and science education with technology and engineering has been published in Turkey, and centers focusing on STEM education in informal settings have been established through private initiatives. In addition, the Ministry of National Education (MEB) has revised the K-12 school curriculum to integrate technology literacy standards into the science education program at the secondary school level (grades 4–8). It has also developed guidelines and curriculum standards for STEM education to encourage mathematics and science teachers to integrate their lessons (Ministry of National Education, 2005, 2006, 2009a,b). However, these innovative initiatives have encountered significant challenges in achieving the expected goals. Foremost among these challenges is the structure of teacher training program; these programs place greater emphasis on theoretical knowledge (subject knowledge or pedagogical theory) than on practical pedagogical content knowledge (Corlu and Corlu, 2010; Kartal, 2011; Özoğlu, 2010). Furthermore, a lack of interdisciplinary coordination and teachers’ inadequate preparation for integrated mathematics and science teaching limit progress (Bulut, 2007).

However, the limitations of science and mathematics teaching programs in Turkey about STEM applications have been highlighted in various studies (Bulut, 2007; Corlu and Corlu, 2010; Corlu, 2012; Ozden, 2007). Research findings indicate that students struggle to transfer mathematical knowledge to new contexts, including science and engineering (Gill, 1999; Harris et al., 2015). The problem here is not only related to mathematical competence or the ability to apply mathematics to other fields; many students also fail to position themselves correctly in the process of transferring mathematical knowledge to another field, which requires strong problem interpretation, the ability to select and apply appropriate tools, adaptability, and clear communication (Britton et al., 2005). Among the significant factors contributing to this integration problem are teacher competence and the incompatibility of curriculum materials (Bulunuz and Ergul, 2001). Recent studies have examined the alignment between the mathematical content in secondary school science textbooks and the secondary school mathematics curriculum (Laçin-Şimşek et al., 2022; Pektaş et al., 2022). The findings revealed that approximately one-third of the questions in science textbooks were inconsistent with the mathematics curriculum. Furthermore, these studies draw attention to broader inconsistencies between science and mathematics teaching programs, indicating that this situation may negatively affect students’ learning and achievement. Therefore, it is recommended that the subject be investigated in greater detail.

### Research design

This study employed a mixed-methods research design to explore how the alignment or misalignment between mathematics and science curricula, as reflected in test items, affects middle school students’ performance in science. Quantitative data were collected through structured multiple-choice assessments, while qualitative insights were gathered via clinical interviews. Together, these methods allowed the researchers to assess students’ achievement patterns and uncover the underlying cognitive and curricular factors contributing to their difficulties, particularly in relation to the mathematical demands of science questions.

### Context and participants

This study was conducted in Turkey, where the education system is centralized and standardized at the national level. At the primary education level (grades 1–8), all pupils follow a common mathematics curriculum developed by the Ministry of National Education (MEB) and defined in official curriculum guides (Ministry of National Education, 2024). These guides determine the scope, sequence, and teaching approaches for each grade level and form the basis for the mandatory mathematics textbooks in all public and private schools. These textbooks are officially reviewed and approved by the Council of Education.

Due to the standardization of curricula and teaching materials across the country, a suitable sampling method was chosen. The sampling process was carried out in two stages. In the first stage, eleven public schools in a coastal city in northern Turkey (five urban schools, six rural schools) were selected to balance school context, student achievement levels, and socio-economic characteristics. One science teacher from each school volunteered to assist with the student recruitment process, and initially 635 students agreed to participate. In the second stage, data from students who provided incomplete or inconsistent responses were excluded. The final sample consisted of 620 students: 344 fifth- and 276 sixth-graders. Table 1 presents the distribution of students by grade level, gender, and school. Prior to the commencement of the study, informed consent was obtained from all participants. Ethical considerations were strictly observed in accordance with institutional and international research ethics standards throughout the design, implementation, and reporting phases.

**Table 1:** Sample sizes for each school.

School	Fifth grade		Sixth grade	
	Female	Male	Female	Male
Urban 1	16	19	16	13
Urban 2	14	22	14	15
Urban 3	18	15	16	15
Urban 4	19	14	13	14
Urban 5	18	17	15	13
Rural 1	15	17	12	14
Rural 2	14	13	14	13
Rural 3	16	11	15	11
Rural 4	15	14	13	12
Rural 5	12	16	15	13
Rural 6	15	14	–	–

### Instruments and data collection

The data were collected using two instruments. The first one is the Multiple-Choice Science Test (MCST), which examines the effect of mathematical connectedness on students’ science achievement for each grade level. These tests were designed to systematically compare student performance on science questions with varying levels of mathematical alignment. Each MCST consisted of eight questions organized into four pairs. Each pair included:

- Mathematically disconnected science question (CDQ): A question requiring mathematical knowledge not covered in the students’ current mathematics curriculum.
- Mathematically connected science question (CCQ): A question that measures the same science learning objective but is reworded using mathematical knowledge appropriate to the class level.

The development process of the MCST was carried out using the study by Pektaş et al. (2022), which examined the alignment between the mathematical content in science textbooks and the national mathematics curriculum. This study showed that the misalignment was particularly high in the fifth (49%) and sixth (39%) grade levels and revealed a significant curriculum gap. Based on these findings, science questions involving mathematical demands were systematically selected from textbooks and converted into the matched CDQ–CCQ format. Table 2 presents the mathematical and science content coverage of the items included in the test. The draft versions of the MCSTs were submitted for expert review to ensure the validity, clarity, and comparability of each item pair. Experts in science and mathematics education evaluated the items for content accuracy, curricular alignment, and fairness. Revisions were made accordingly to improve the precision of alignment between each item’s mathematical demands and the students’ actual curricular exposure.

**Table 2:** Mathematics and science content coverage for the test items.

Question ID	Fifth grade level		Sixth grade level	
	Science content	Mathematics content	Science content	Mathematics content
CCQ1	Measuring force	Natural numbers	Solar system	Bar graphs
CDQ1	Measuring force	Proportion/ratio	Solar system	Curve graphs
CCQ2	Phases of the moon	NA	Enzymes	NA
CDQ2	Phases of the moon	The area under a curve	Enzymes	Curve graphs
CCQ3	Melting point	Natural numbers	Speed	Natural numbers
CDQ3	Melting point	Integers	Speed	Line graphs
CCQ4	Reflection of light	Angle measures	Mass and density	Bar graphs
CDQ4	Reflection of light	Constructing altitude	Mass and density	Line graphs

The second data collection tool was audio recordings of semi-structured interviews. To examine students’ reasoning processes and the difficulties they encounter in greater depth, semi-structured clinical interviews were conducted with a subgroup of students who completed the MCST. These interviews were designed to reveal (i) the level of student understanding of science concepts as measured by questions, (ii) the role of mathematical content in facilitating or hindering science-related reasoning, and (iii) students’ perceptions of the connections between mathematics and science in their learning experiences. Each interview lasted approximately 20–30 min and was conducted individually in a quiet environment to minimize distractions. All interviews were audio-recorded with the participants’ consent and transcribed digitally. A semi-structured protocol was followed during the interviews; predetermined and follow-up questions were asked to encourage participants to elaborate on their reasoning processes. Throughout the interviews, students were asked to explain how they approached selected MCST questions, discuss their difficulties, and describe in detail how they used mathematical concepts when solving science-related problems.

## Data analysis

Responses from the MCST were analyzed using descriptive statistics to summarize student performance on CCQs and CDQs. To determine whether performance differences between item types were statistically significant, first, the distribution of the data was examined, and it was determined that there were no outliers. A Kolmogorov–Smirnov normality test on item scores revealed that the scores do not meet the assumption of normal distribution. It was also observed that the score distributions for the scales represent continuous data measured at an interval level. The assumptions for conducting parametric tests include the independence of the two samples (groups), the measurement of dependent variables at an interval or ratio scale, and the fulfillment of normality and homogeneity assumptions (Köklü et al., 2007). Since these assumptions were not met, non-parametric statistical techniques were used in the analyses. These analyses enabled the researchers to examine the impact of curriculum alignment on science achievement across grade levels.

Interview transcripts were subjected to thematic analysis. Coding focused on identifying recurring challenges students experienced with CDQs, including:

- Use of unfamiliar or untaught mathematical procedures,
- Misinterpretation of questions due to mathematical complexity,
- Inability to apply science knowledge when mathematical barriers were present.

These themes provided insight into the mechanisms through which curricular misalignment affected students’ science performance and offered explanatory support for the quantitative findings. Particular importance was given to the consistency between the students’ verbal explanations in the interviews and their responses on the MCST. This analysis revealed students’ difficulties when responding to CDQ items and provided contextual explanations for the quantitative patterns observed in the MCST results.

## Results

Table 3 presents the results of the Wilcoxon Signed Ranks Test comparing the achievement levels of fifth-grade students on two types of science questions: CCQs, which align with students’ expected mathematical knowledge, and CDQs, which require mathematical skills beyond the grade-level curriculum.

**Table 3:** Wilcoxon signed-rank test for the difference between the scores of fifth-grade students.

Tested pair	Question pairs	*N*	*X–*	*S*	Mean rank	Sum of rank	*Z*	*p*
Overall	CCQs	344	2.58	1.14	143.61	32599.50	−11.36	0.000*
	CDQs	344	1.52	1.14	92.69	3985.50		
Pair 1	CCQ1	322	0.78	0.42	64.50	5934.00	−4.95	0.000*
	CDQ1	322	0.60	0.49	64.50	2322.00		
Pair 2	CCQ2	321	0.82	0.39	104.00	20176.00	−12.58	0.000*
	CDQ2	321	0.25	0.44	104.00	1352.00		
Pair 3	CCQ3	326	0.46	0.50	76.00	7524.00	−3.83	0.000*
	CDQ3	326	0.32	0.47	76.00	3952.00		
Pair 4	CCQ4	323	0.61	0.49	80.50	8694.00	−4.43	0.000*
	CDQ4	323	0.43	0.50	80.50	4186.00		

*p < 0.001.

The analysis reveals a statistically significant difference between the mean ranks of students’ scores on CCQs and CDQs, as indicated by *Z* = −11.36, *p* < 0.001. This result suggests that fifth-grade students performed significantly better on science questions that were aligned with their mathematical knowledge. In contrast, their performance declined on questions requiring mathematical understanding not yet covered in the curriculum. These findings indicate that a lack of curricular alignment between mathematics and science may hinder students’ ability to successfully engage with certain types of science questions.

Each pair of CCQs and CDQs was analyzed separately, and all comparisons yielded statistically significant differences (*p* < 0.001), further reinforcing the overall trend. In Pair 1, students scored slightly higher on CCQ1 (*M* = 0.78) than on CDQ1 (*M* = 0.60), with a significant difference (*Z* = −4.95, *p* < 0.001). Pair 2 revealed the most substantial gap in performance, with students performing markedly better on CCQ2 (*M* = 0.82) compared to CDQ2 (*M* = 0.25), as reflected by a strong *Z*-score (*Z* = −12.58, *p* < 0.001). In Pair 3, students again scored higher on CCQ3 (*M* = 0.46) than on CDQ3 (*M* = 0.32), and this difference was statistically significant (*Z* = −3.83, *p* < 0.001). Similarly, in Pair 4, students demonstrated greater success on CCQ4 (*M* = 0.61) compared to CDQ4 (*M* = 0.43), with a significant difference (*Z* = −4.43, *p* < 0.001). For each question pair, students consistently achieved higher scores when the mathematical content was aligned with their curriculum. The largest performance gap was observed in Pair 2, which focused on learning outcomes related to the Moon’s movement around Earth. These findings suggest that specific mathematical demands in the curriculum-disconnected version of the questions posed a particularly significant challenge for students.

Table 4 presents the statistical findings related to the performance discrepancies among sixth-grade students. The results reveal a consistent pattern with the fifth-grade data, indicating that students performed significantly better on CCQs than on CDQs. Specifically, the mean rank for CCQs was 113.60, compared to 79.19 for CDQs. The Wilcoxon Signed Ranks Test confirmed a statistically significant difference between the two sets of scores (*Z* = −10.14, *p* < 0.001), indicating that students experience greater difficulty when required to apply mathematical concepts beyond the expected curriculum level.

**Table 4:** Wilcoxon signed-rank test for the difference between the scores of sixth-grade students on the question pairs.

Tested pair	Question pairs	*N*	*X–*	*S*	Mean rank	Sum of rank	*Z*	*p*
Total	CCQs	276	2.16	1.32	113.60	20902.00	−10.14	0.000*
	CDQs	276	1.03	1.00	79.19	2534.00		
Pair 1	CCQ1	259	0.69	0.46	67.00	7571.00	−8.06	0.000*
	CDQ1	259	0.33	0.47	67.00	1340.00		
Pair 2	CCQ2	249	0.34	0.47	48.00	3120.00	−3.59	0.000*
	CDQ2	249	0.20	0.40	48.00	1440.00		
Pair 3	CCQ3	261	0.67	0.47	77.00	10241.00	−9.14	0.000*
	CDQ3	261	0.24	0.43	77.00	1540.00		
Pair 4	CCQ4	253	0.54	0.50	56.00	4592.00	−5.03	0.000*
	CDQ4	253	0.33	0.47	56.00	1624.00		

*p < 0.001.

An analysis of individual question pairs further reinforces the overall trend. In each pair, students demonstrated higher success rates on CCQs than on CDQs, with statistically significant differences across all comparisons. For example, in Pair 1, students performed substantially better on CCQ1 (*M* = 0.69) than on CDQ1 (*M* = 0.33), as indicated by a *Z*-score of −8.06 (*p* < 0.001). A similar but even more pronounced difference was found in Pair 3, which addressed the role of enzymes in chemical reactions; students scored significantly higher on CCQ3 (*M* = 0.67) than on CDQ3 (*M* = 0.24), with a *Z*-score of −9.14 (*p* < 0.001). Although Pair 2 exhibited the smallest difference, the result remained statistically significant (*Z* = −3.59, *p* < 0.001), suggesting that although some mathematical prerequisites may have been partially met, students continued to face challenges.

When comparing these results with the fifth-grade findings, a consistent trend emerges: students at both grade levels perform significantly worse on questions that require advanced mathematics skills. However, there are subtle differences in performance patterns. In the fifth-grade data, the most substantial gap appeared in Pair 2 (*Z* = −12.58, *p* < 0.001), whereas in sixth grade, the greatest challenge shifted to Pair 3 (*Z* = −9.14, *p* < 0.001). This suggests that as students advance, certain types of mathematical reasoning (such as interpreting the slope of a line graph, finding the area under a curve, and making inferences from curved graphical representations) may become more demanding within the science curriculum. Additionally, while fifth graders had a slightly higher overall CCQ mean (2.58) compared to sixth graders (2.16), both groups showed a significant drop in performance on CDQs, reinforcing the notion that the misalignment between the math and science curricula continues into higher grades.

**Figure 1:**
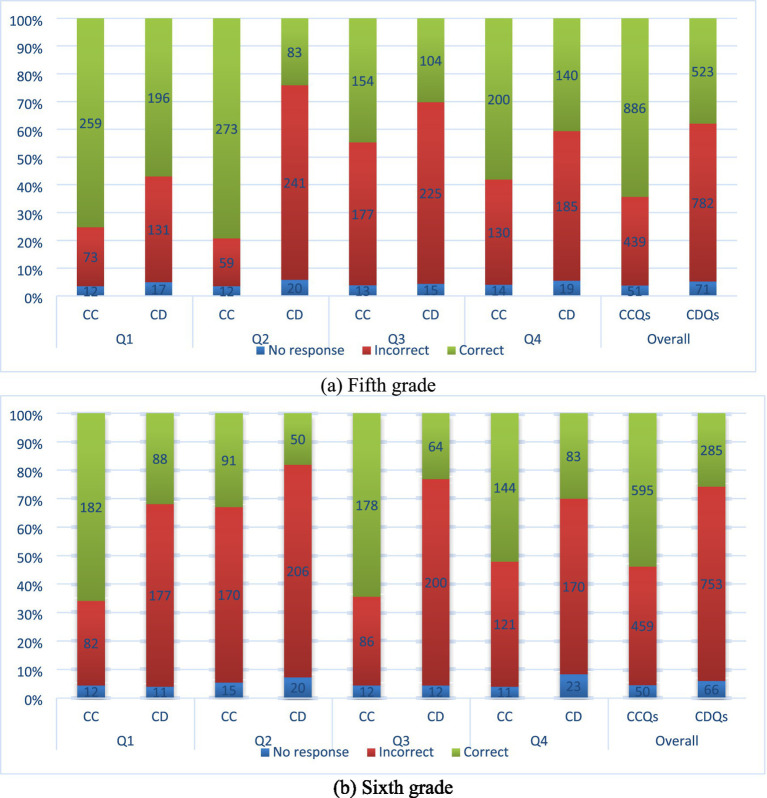
Students’ performance on the science questions with the math curricula connection (CC) and math curricula disconnection (CD) for fifth and sixth grade students.

In this segment, we offer detailed, qualitative discourse on the manner in which mathematics curriculum alignment within the scientific domain is reflected in student achievement, thereby facilitating an in-depth and comprehensive comprehension of the statistically significant findings outlined above. As illustrated in Figure 1, the distribution of students’ performance on mathematics-connected and math-disconnected science questions is presented. At the fifth-grade level, students demonstrated higher performance in the CCQs, as evidenced by the higher rates of false responses and the tendency to leave the CDQs blank (Figure 1a). For instance, approximately 82% of the fifth-grade students correctly answered CCQ2, which inquired about the moon’s phases. This rate decreased to approximately 25% for CDQ2. Which evaluated curve graphs using mathematical skills. Analogous results were observed for sixth-grade students (see Figure 1b). Sixth graders demonstrated a higher proficiency in answering CCQs, but exhibited greater difficulty in responding to CDQs, particularly those pertaining to the solar system and velocity (question pairs 1 and 3, respectively). Notably, more than half of the students provided accurate responses when the questions employed connected mathematics objectives (CCQ1 and CCQ3). However, the rate of correct solutions decreased for questions with disconnected mathematics objectives. This decline was particularly evident in questions that employed mathematics objectives, not attaining grade level, such as evaluating graphs and understanding scope (CDQ3 at the sixth grade level).

**Figure 2:**
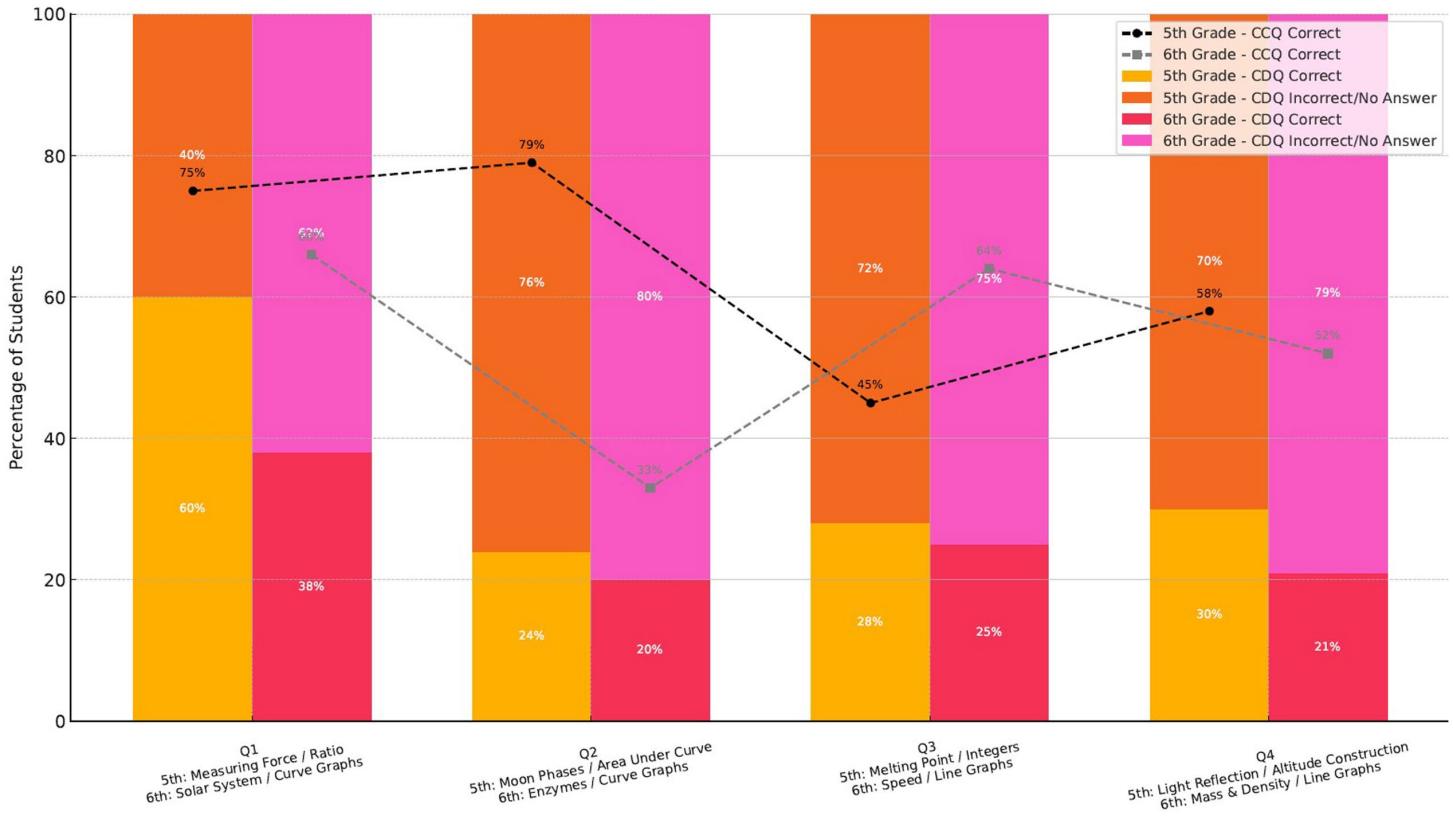
The impact of mathematical alignment on student performance in science questions.

In the assessment of students’ success, a salient question emerged: How did the students’ status of success, defined by their accurate responses to questions requiring proficient mathematical aptitude, in a specific science outcome, shift when considering mathematical competencies within the same science outcome but not yet attained? Figure 2 elucidates the findings concerning the success status of students who correctly answered each CCQ in the CDQs belonging to the same outcome. The analysis of student performance on CCQs and CDQs reveals a consistent pattern: students perform significantly better when the mathematical demands of a science item align with their grade-level mathematics curriculum. Across all four question pairs, both fifth- and sixth-grade students showed markedly higher accuracy on CCQs, with sharp declines in performance on the corresponding CDQs. This suggests that students’ success in science assessments is not solely dependent on their conceptual understanding of scientific content but also on whether they have the mathematical tools to access the question.

**Figure 3:**
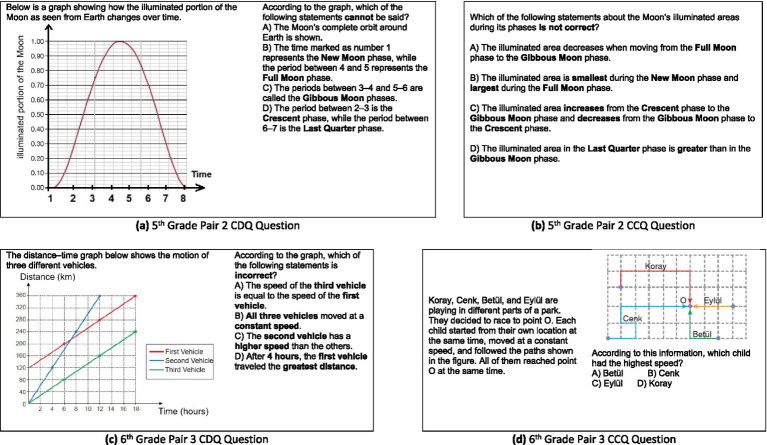
CDQ and CCQ questions for 5th-grade Pair 2 and 6th-grade Pair 3.

At the fifth grade level, this trend is most evident in Question Pairs 2 (see Figures 3a,b), which required the science knowledge of the phases of the Moon. For fifth-grade students, the mathematics disconnected item (CDQ2, Figure 3a) involved identifying lunar phases using a graph of illuminated area across the moon’s cycle. Although students could articulate scientific ideas like the progression from new moon to full moon, they struggled to interpret the shape of the graph. One student, for instance, explained the sequence of phases accurately but hesitated when asked to compare brightness based on curve height. She responded, “I do not know how much is ‘half’ on the graph… It’s like a hill. I do not know the numbers.” Her difficulty stemmed not from gaps in her science knowledge, but from the mathematical abstraction of interpreting area under a curve, a concept not typically taught at the fifth-grade level.

A similar situation was observed in the sixth-grade regarding the CDQ2 (see Figures 3c,d), which presented an enzyme reaction diagram comparing two curves. The student interviewed showed a clear understanding of the scientific principle that enzymes reduce activation energy. However, when asked to determine which curve represented the enzyme-catalyzed reaction, the student responded, “I know the one with lower energy is the one with the enzyme, but I do not know how to compare the graphs… Maybe if they gave numbers, it would be easier.” This again highlights how the absence of numerical data, combined with the need for visual estimation and curve interpretation, creates a barrier rooted in math, not in science.

The issue becomes even more pronounced in CDQ3 for sixth graders, which involved analyzing a distance-time graph to determine the speed of different vehicles. The interviewed student demonstrated a strong understanding of speed as distance over time, but was unable to connect this with the slope of the graph. When prompted, she said, “I’ve heard of slope… but we have not really learned that in math yet.” This illustrates how students’ inability to interpret slope or interpolate between points prevents them from fully accessing the scientific reasoning required by the item.

Collectively, these qualitative accounts support the quantitative finding that mathematical misalignment disproportionately affects students’ science performance. Students often possess the relevant science knowledge but are unable to apply it when the mathematical representation or reasoning exceeds their instructional level. These findings reinforce the importance of curricular coordination in integrated science and mathematics education and raise questions about fairness in science assessment. When test items rely on mathematical constructs that have not yet been taught, they may measure mathematical readiness rather than science understanding. For both instruction and assessment design, this underscores the critical need for intentional alignment between mathematics and science curricula.

## Discussions

The current study aimed to examine how the alignment or misalignment of mathematical content in science textbooks affects secondary school students’ science achievement. The findings indicate that students’ science achievement is significantly influenced by the degree of mathematical consistency embedded in textbook-based science questions. Quantitative and qualitative data reveal that students perform better on science questions when the necessary mathematical concepts have been previously taught in their current mathematics curriculum. This highlights the critical importance of interdisciplinary consistency between mathematics and science and aligns with previous research indicating that inconsistent curriculum content leads to cognitive difficulties (Gustin and Corazza, 1994; Wang, 2005). Researchers advocating integrated teaching argue that using mathematical terms and tools facilitates students’ understanding of scientific concepts (Basista and Mathews, 2002). These findings also support studies showing that fragmented curricula hinder students’ interdisciplinary knowledge transfer (Czerniak et al., 1999; Schuchardt and Schunn, 2016). For example, Schuchardt and Schunn (2016) found that students struggled with scientific reasoning tasks when mathematical principles were taught independently. Similarly, Czerniak et al. (1999) reported that integrated approaches enhanced student engagement and conceptual understanding in science and mathematics. These findings suggest that curriculum developers and educators should prioritize inter-curricular coherence and consistency to enhance interdisciplinary learning and achievement.

Importantly, mathematical inconsistency lowers students’ science performance and hinders their ability to transfer scientific knowledge. Even if students have sufficiently understood scientific concepts, they may fail to demonstrate this knowledge when confronted with unfamiliar mathematical representations or reasoning processes (Gill, 1999; Harris et al., 2015). This problem is not limited to mathematical competence or the capacity to adapt mathematics to other disciplines; it also reflects the difficulties students experience in positioning themselves correctly during transferring mathematical knowledge and strategies to another field, such as applying mathematical reasoning within scientific contexts (Britton et al., 2005). Clinical interviews have also revealed that students experience cognitive overload when scientific content is presented through representations that require mathematical skills not yet taught, such as interpreting slopes or curves. These findings confirm that the integration of mathematics into science teaching must be deliberate, pedagogically structured, and carefully designed, particularly in the context of assessments.

Notably, integration issues are influenced by factors such as teacher competence and the incompatibility of curriculum materials; this situation has also been emphasized in the research of Bulunuz and Ergul (2001). Pektaş et al. (2022), who examined the compatibility of science and mathematics curriculum elements, found that many of the questions in science textbooks contained mathematical content incompatible with the secondary school mathematics teaching program. The study also drew attention to broader incompatibilities between science and mathematics curricula and emphasized the need to investigate how students cope with such questions. In this vein, the findings of our study also show that inconsistencies in interdisciplinary curriculum elements (e.g., exam content) negatively affect student performance. The lack of alignment between science and mathematics curricula hinders students’ understanding of the relationships between the two disciplines and weakens their knowledge transfer skills. Moderate correlations between mathematics and science achievement indicate that strategic mathematical knowledge is necessary for success in science learning (Gustin and Corazza, 1994; Wang, 2005). Proponents of integrating science and mathematics courses note that using mathematical terms and tools facilitates students’ understanding of scientific concepts (Basista and Mathews, 2002).

It is essential to align the science curriculum with the mathematics teaching program to develop students’ competence in using mathematics in science contexts and, at the same time, to deepen their understanding of mathematics. This recommendation is also supported by the National Research Council (1996, p. 214). Furthermore, Berlin and White (2010) argue that integrated teaching programs positively influence students’ attitudes towards mathematics and science. Making the curriculum consistent will also enable teachers to develop interdisciplinary teaching strategies, contributing to students’ learning in both areas. In conclusion, addressing the inconsistencies in science and mathematics curricula is critical to improving students’ success in both disciplines, and this must be reflected in teaching practices and curriculum design.

## Implications

The findings of this study highlight the pressing need to strengthen mathematics and science teacher preparation programs through a STEM-integrative approach that directly addresses curricular incoherence and disciplinary isolation. Developing such programs in colleges of education is critical for equipping pre-service teachers with the competencies required to translate theoretical knowledge into applicable, real-world models across education, technology, and industry. Integrative STEM frameworks foster teachers’ capacity to design interdisciplinary lessons that connect mathematical reasoning with scientific inquiry, use data analysis and modeling as tools for conceptual exploration, and engage learners in authentic, problem-based activities. This approach cultivates creativity, collaboration, and systems thinking—skills necessary to prepare students for a knowledge economy increasingly shaped by innovation and digital transformation (Rodríguez et al., 2024; Alrwaished, 2024; Wu et al., 2024). By embedding project-, design-, and inquiry-based methodologies into teacher education, institutions can produce reflective practitioners capable of bridging theory and practice while reinforcing coherence across mathematics and science curricula (Navy and Kaya, 2020; Milner-Bolotin, 2018).

Equally important, systemic alignment between teacher education programs, institutional policies, and national standards is essential to sustain these innovations. Policymakers and educational leaders should promote evidence-based, interdisciplinary professional development that enables teachers to continuously refine their integrative instructional practices. Establishing frameworks for collaboration between mathematics and science departments, designing coherent assessment systems that capture students’ cross-disciplinary understanding, and embedding technology-enhanced learning opportunities will further enhance the efficacy of STEM-integrated education (Silva-Díaz et al., 2023; Yilmaz, 2022; Berisha and Vula, 2023). When effectively implemented, such programs transcend traditional disciplinary boundaries, empowering educators to become agents of transformation who link abstract theory to social, technological, and economic innovation. In this regard, STEM-integrative teacher preparation should not be viewed merely as an academic enhancement but as a strategic investment in developing educators capable of guiding future generations toward sustainable, interdisciplinary, and innovation-driven futures.

## Data Availability

The raw data supporting the conclusions of this article will be made available by the authors, without undue reservation.
